# Investigation of antifouling properties of polypropylene/TiO_2_ nanocomposite membrane under different aeration rate in membrane bioreactor system

**DOI:** 10.1016/j.btre.2019.e00414

**Published:** 2019-12-31

**Authors:** Habib Etemadi, Milad Fonouni, Reza Yegani

**Affiliations:** a Department of Polymer Science and Engineering, University of Bonab, Bonab, Iran; b Faculty of Chemical Engineering, Sahand University of Tehnology, Tabriz, Iran; c Membrane Technology Research Center, Sahand University of Technology, Tabriz, Iran

**Keywords:** Membrane bioreactor, Aeration rate, Polypropylene membrane, Antifouling

## Abstract

This paper presents the results of aeration rate effect on antifouling properties of polypropylene (PP)/TiO_2_ nanocomposite membrane in membrane bioreactor (MBR) system in order to oil refinery wastewater treatment. For this purpose, three levels of aeration rate with specific aeration demand per membrane area (SADm) of 0.5, 1, and 1.5 m^3^/m^2^h was used. According to the obtained results, PP nanocomposite membrane showed high hydrophilicity, porosity, and high flux compared to neat PP membrane. Also, either low or high aeration rate had a negative influence on permeability and antifouling properties of neat PP and nanocomposite membranes. The analysis of fouling mechanism for both membranes based on Hermia’s model revealed that the cake formation is dominant mechanism for lower aeration rate while by increasing aeration rate, all models couldn’t predict experimental data. Meanwhile, by increasing aeration rate, chemical oxygen demand (COD) removal for activated sludge and both membranes decreased and increased, respectively.

## Highlights

• PP/TiO_2_ membrane was fabricated via thermally induced phase separation (TIPS) method.
• Effect of aeration rate was investigated on antifouling properties of both membranes in MBR system.
• Fouling properties of membranes under different aeration rates were analyzed using Hermia’s models.
• Nanocomposite membrane showed high antifouling compared to neat membrane.
• Increasing in high aeration rate resulted in sludge floc breakage as well as pore blockage.

## Graphical abstract

**Figure:**
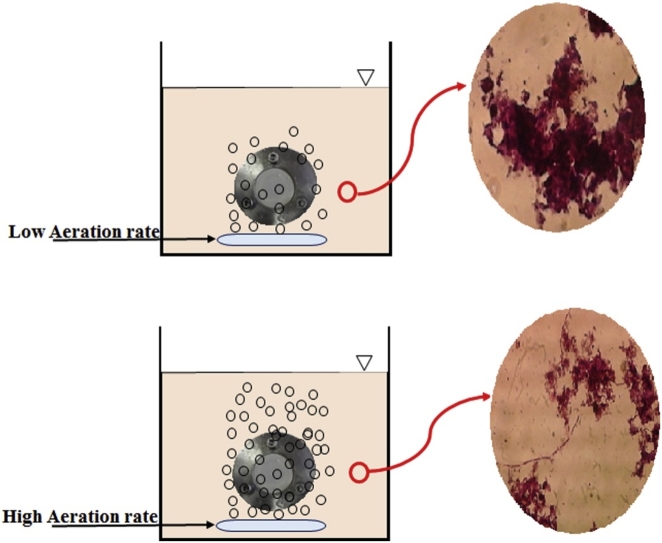

## 1 Introduction

Today water scarcity is a serious problem all over the world due to the increasing in population and the expansion of industry activities [1]. Therefore, it seems that wastewater treatment and reuse are necessary. Among several wastewater reuse and recycling processes, membrane-based technologies show great potential to overcome water scarcity [2]. In this case, membrane bioreactor (MBR) technology widely used for the treatment of various municipal and industrial wastewater, because of small footprint demand and high quality of effluent compared to other conventional wastewater treatment systems [3,4]. However, membrane fouling and prohibitively costly compared with the more established conventional technologies is the major problem impeding the widespread adoption of MBR to full-scale plants [[5], [6], [7]].

According to the literature, two strategies including of operational conditions and membrane modification was widely used to improvement in antifouling properties of membrane in MBR system. Among operational conditions, many studies have been focused on the effect of aeration rate on the membrane fouling. It is well known that the aeration intensity strongly impacts the mixed liquor organic matter fractions and correspondingly influences the membrane fouling rate [8]. Ivanovic et al. [9] showed that the relationship between sufficient aeration to minimize membrane fouling. Also, they proposed an approach to define optimal operating conditions with respect to aeration rates. Meng et al. [10] investigated the effect of aeration rate on membrane fouling in submerged MBR. They concluded that aeration had a positive effect on cake layer removal, but pore blocking became severe as aeration intensity increased to 800 L/h. In other words, under low aeration rate, foulants on the membrane surface was not removed effectively, while, high aeration could induce a severe breakage of sludge flocs.

Among all modification methods in membrane preparation, nanocomposite membranes demonstrate promising performances and are predicted to gather the intrinsic properties of both polymeric and inorganic membranes and give interesting advantages of the hybrid membrane such as great thermal and chemical resistances, good antifouling and separation performances, and excellent adaptation to the severe operating conditions [11]. In this regard various nanoparticles were used in order to improvement in antifouling properties of membrane in MBR systems.

On the other hand, using low cost membrane such as polypropylene (PP), can reduce the MBR costs. PP are good candidates for preparation of membrane due to high mechanical strength, high chemical stability, thermal resistance and low cost [11,12]. Therefore, PP is a very promising material for separating membranes. Although the prepared PP membrane exhibit many advantages, it still has several disadvantages, such as low porosity, poor hydrophilicity and high fouling [12,13]. The disadvantages reduce the water flux of the PP membrane and limit applications of this membrane in wastewater treatment applications. Therefore, it seems that PP membrane modification is essential in order to using in MBR system. For this purpose, as mentioned above, incorporation of hydrophilic nanoparticles into polymer matrix is one of the effective methods to enhance the membrane antifouling properties. Among different inorganic nanoparticles, titanium dioxide (TiO_2_) has received most of the attention because of its unique specifications such as stability under harsh conditions, commercial availability and easiness of preparation.

In our previous work [13], PP/TiO_2_ nanocomposite membrane was prepared via thermally induced phase separation (TIPS) method and it was tested in MBR system. However, in the current work, the effect of aeration rate on antifouling properties of PP/TiO_2_ nanocomposite membrane was investigated using oil refinery wastewater influents obtained from Tabriz Oil Refinery Co. in MBR system. In this case, the fouling mechanism as well as antifouling performance of both fabricated membrane (neat and nanocomposite membranes) was investigated.

## 2 Experimental

### 2.1 Materials

Isotactic PP with commercial grade (EPD60R) was supplied from Arak Petrochemical Co., Iran. The melt flow index of PP was 0.35 g/10 min. TiO_2_ nanoparticles (particle size of ca. 21 nm) were purchased from Sigma- Aldrich (Germany). Mineral oil as diluent, acetone as extracting agent and Irganox 1010 as heat stabilizer were purchased from Acros Organics (Belgium), Merck (Germany) and Ciba Co. (Switzerland), respectively. All materials were used as-received unless otherwise described.

### 2.2 Membrane preparation

The neat PP and nanocomposite membrane was fabricated by TIPS method using a sealed glass vessel kept in silicone oil bath. A certain amount of TiO_2_ nanoparticles (0.75 wt.%) with Irganox (1 wt% of solid phase) were dispersed into 60 g of mineral oil using bath sonication (Woson, China) for 60 min. Then, PP was added to the diluent-TiO_2_ suspension and melt blended at 170 ℃ for 90 min. The solution was then allowed to degas for 30 min and cast on a preheated glass sheet using a doctor blade with the film thickness of 250 μm. The plate was immediately quenched in the water bath (30 ± 3 °C) to induce phase separation. The membrane was then immersed in acetone for 24 h to extract its diluent.

### 2.3 Membranes characterization

The microscopic morphology of the neat PP and nanocomposite membranes was characterized by scanning electron microscope (SEM) (VEGA3, TESCAN). The hydrophilic properties of membranes was evaluated by measuring the contact angle between membrane surface and water droplet using a goniometer (PGX, Thwing-Albert Instrument Co., USA). All reported data of contact angle are the average of five different tests from each membrane sample.

Membrane porosity was measured using gravimetric method. In this method, PP membranes were immersed in i-butanol for 24 h and then immediately weighed after removing i-butanol from the surface. The porosity was calculated using the following equation [12]:

(1) $$\varepsilon (\%)=\frac{(W_{\mathrm{wet}}-W_{\mathrm{dry}})/D_{i}}{(W_{\mathrm{wet}}-W_{\mathrm{dry}})/D_{i}+W_{\mathrm{dry}}/D_{p}}\times \mathrm{100}$$

where W_dry_ is the initial membrane weight, W_wet_ is the membrane weight after 24 h immersion in i-butanol, D_p_ and D_i_ are the density of PP (0.91 g/cm^3^) and i-butanol (0.8 g/cm^3^), respectively.

Tensile strength of the membranes was analyzed via tensile test machine (STM-5, SANTAM) at an extension rate of 50 mm/min. At least three measurements were carried out and the mean value for each case was reported.

The tortuosity (τ) of the membrane was determined using Eq. (2) [14]:

(2) $$\tau =\frac{{\left(2-\varepsilon \right)}^{2}}{\varepsilon }$$

### 2.4 MBR set-up

**Fig. 1:**
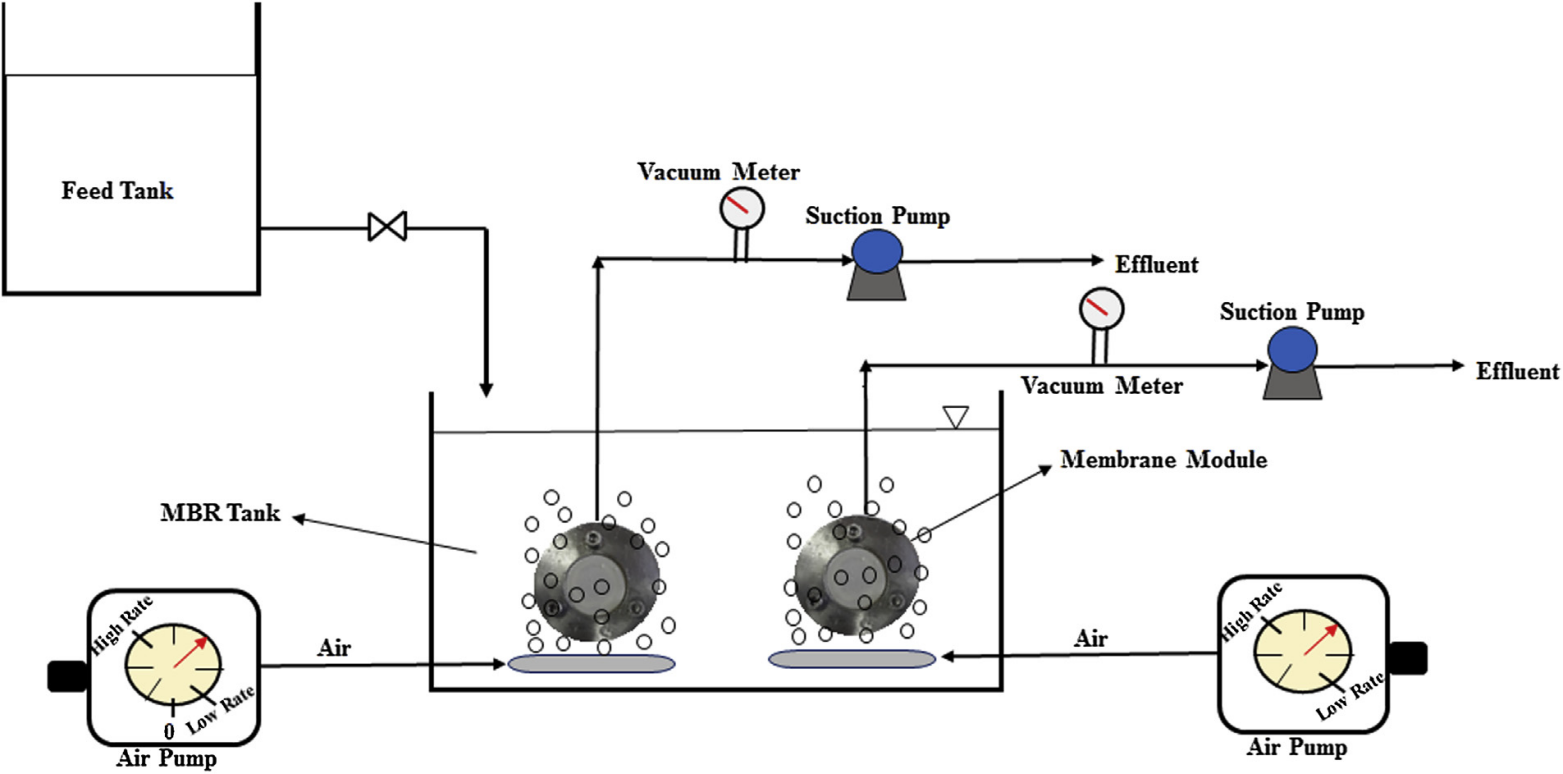
Schematic of MBR setup in this study.

In this study, a lab-scale submerge MBR (12 L working volume) was used. The flat sheet membrane modules had a volume of 50 mL and an effective membrane filtration area of 14.7 cm^2^. Fig. 1 shows flat sheet modules submerged in the MBR test system. An air diffuser was installed beneath the membrane module to provide dissolved oxygen as well as efficient agitation of activated sludge in the MBR. Transmembrane pressure (TMP) was maintained constant at 0.1 bar. Mixed liquor suspended solid (MLSS) concentration was about 7000 mg/l. Hydraulic retention time (HRT) and sludge residence time (SRT) were maintained at 24 h and 20 days, respectively.

Real wastewater with chemical oxygen demand (COD) of 178 mg/l was supplied from Tabriz Oil Refinery Company (TZ.O.R.C), Tabriz, Iran. The literature showed that air injection reduced fouling in an submerged MBR up to a critical flow rate corresponding to a specific aeration demand per membrane area (SADm) of 0.25 m^3^/m^2^ h [15,16]. Therefore, in this study, the lower value of SADm was corresponding to 0.5 m^3^/m^2^ h. The effect of aeration rate on the membrane fouling was examined, and in this case, SADm was selected for three different rates; 0.5, 1, and 1.5 m^3^/m^2^ h.

### 2.5 Antifouling performance of membranes

Antifouling performance of neat PP and PP/TiO_2_ (0.75 wt.%) membranes were evaluated by filtrating activated sludge. After pure water flux tests (J_w1_, L/m^2^h), filtration experiments were carried out for 360 min at 0.1 bar and the flux for activated sludge (J_AS_, L/m^2^h) was measured. Then the membrane was taken out for simple cleansing under running deionized water and the pure water flux of cleaned membranes J_w2_ (L/m^2^h) was measured again. The flux recovery ratio (FRR) was calculated as follows:

(3) $$\mathrm{FRR}(\%)=\frac{J_{\mathrm{w2}}}{J_{\mathrm{w1}}}\times \mathrm{100}$$

Furthermore, the antifouling property of membranes was also evaluated by the total fouling ratio (TFR), reversible fouling ratio (RFR) as well as irreversible fouling ratio (IFR) according to the following equations [4,17]:

(4) $$\mathrm{RFR}(\%)=\left(\frac{J_{\mathrm{w2}}-J_{\mathrm{AS}}}{J_{\mathrm{w1}}}\right)\times \mathrm{100}$$

(5) $$\mathrm{IFR}(\%)=\left(\frac{J_{\mathrm{w1}}-J_{\mathrm{w2}}}{J_{\mathrm{w1}}}\right)\times \mathrm{100}$$

(6) $$\mathrm{TFR}(\%)=\mathrm{RFR}(\%)+\mathrm{IFR}(\%)=\left(\frac{J_{\mathrm{w1}}-J_{\mathrm{AS}}}{J_{\mathrm{w1}}}\right)\times \mathrm{100}$$

COD removal was estimated by measuring COD of efﬂuent (COD_E_) and inﬂuent (COD_I_) based on absorbance method as described elsewhere [18] and using the following equation [18]:

(7) $$CODRemoval\left(\%\right)=(1-\frac{COD_{E}}{COD_{I}})\times 100$$

### 2.6 Analysis of fouling mechanisms

According to Hermia’s model, under constant pressure filtration condition, four fouling mechanisms blamed for flux decline could be explained using the following mathematical equation [19]:

(8) $$\frac{d^{2}t}{dV^{2}}=k{\left(\frac{\mathrm{dt}}{\mathrm{dV}}\right)}^{m}$$

where t (h) is filtration time, V (m^3^) is the filtrate volume, k is the resistance coefficient and specific formulation of these fouling mechanisms could be characterized with different values of m: m = 0 for cake filtration, m = 1 for intermediate blockage, m = 1.5 for standard blockage and m = 2 for complete blockage [20]. Using the flux expression (Eq. (9)), the flux decline expression can be expressed by Eq. (10):

(9) $$J=\frac{1}{A}\frac{\mathrm{dV}}{\mathrm{dt}}$$

(10) $$\frac{\mathrm{dJ}}{\mathrm{dt}}=-kA^{2-m}J^{3-m}$$

In Eqs. (9) and (10), A is the effective membrane area (m^2^).

## 3 Results and discussion

### 3.1 Membrane morphology

**Fig. 2:**
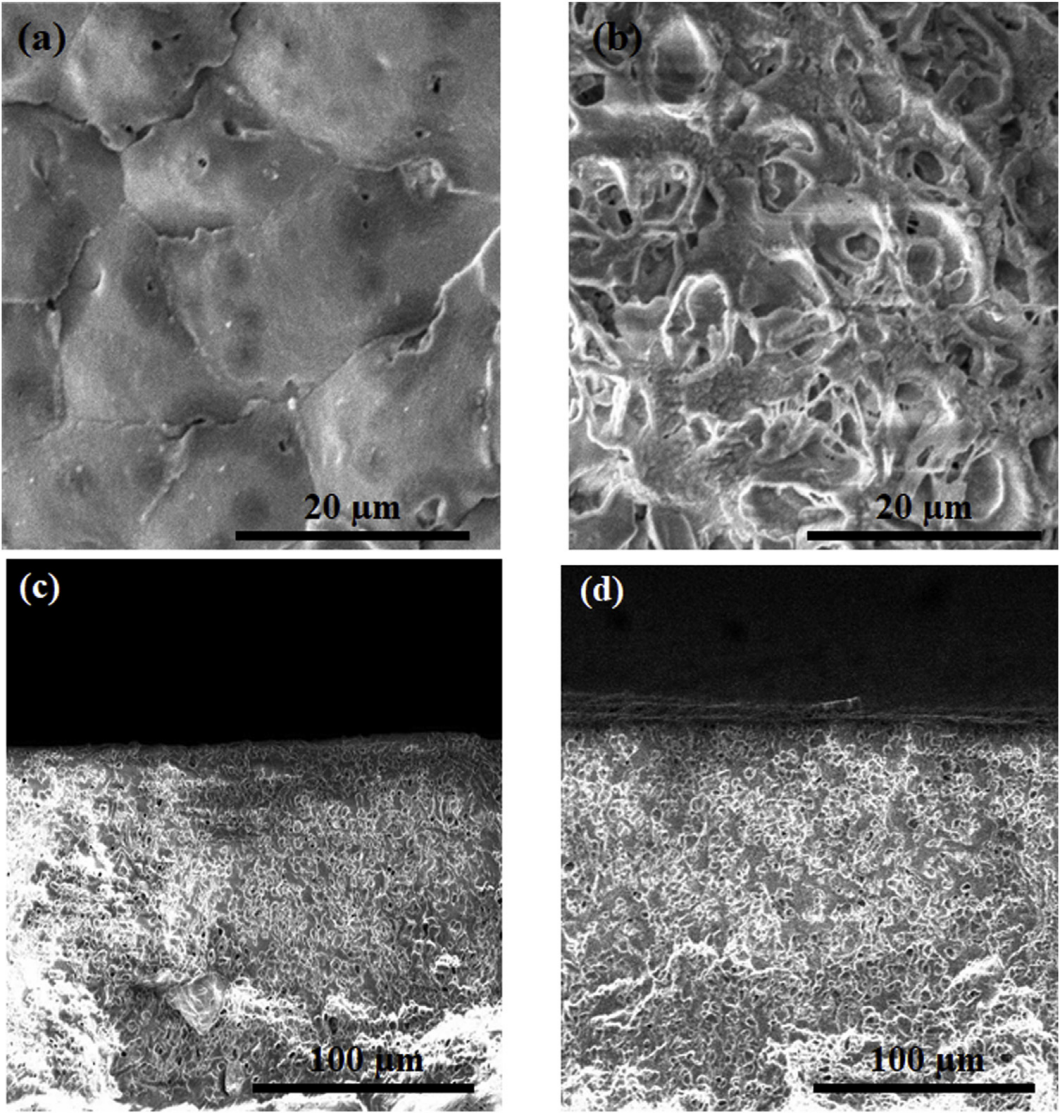
SEM images of membrane surface of (a) neat PP, (b) PP/TiO_2_ and cross section of (c) neat PP and (d) PP/TiO_2_ membranes.

SEM images of surface of neat PP and PP/TiO_2_ (0.75 wt.%) membranes, is shown in Fig. 2a and b. By addition of TiO_2_ nanoparticles the nucleation density and porosity increased in PP nanocomposite membrane when compared with neat PP membrane. In other words, due to the fact that TiO_2_ nanoparticles acted as the crystal nuclei and a certain amount of TiO_2_ dosage could increase the number of spherulites, decrease the size of spherulites and caves between the spherulites and make spherulites more uniform. Similar results was found in elsewhere [11]. Fig. 2a and b also show that the addition of TiO_2_ nanoparticles increased the number and the size of pores in the surface of membrane. This may be due to the heterogeneous nucleation effect of TiO_2_ nanoparticles.

Fig. 2c and d show the SEM images of cross section of membranes. From the SEM images of cross section it can be seen that the both membrane has sponge like porosity and symmetric structure.

### 3.2 Hydrophilicity, porosity and tensile strength of membranes

The results of contact angle measurement which is responsible for membranes hydrophilicity, are shown in Table 1. It can be seen that the hydrophilicity of PP membrane improved with the addition of TiO_2_ nanoparticles in which the water contact angle decreases. The relatively higher hydrophilicity was found for PP/TiO_2_ nanocomposite membranes due to the presence of hydroxyl functional groups on the TiO_2_ nanoparticle surface [21].

**Table 1:** Water contact angle, porosity, tortuosity and tensile strength of neat PP and nanocomposite membranes.

Membrane type	Water contact angle (°)	Porosity (%)	Tortuosity	Tensile strength (MPa)
Neat PP	122.2	31.5	9.0	4.1
PP/TiO2 (0.75 wt.%)	106.0	50.7	4.4	4.7

Overall porosities of the fabricated membranes are presented in Table 1. By addition of 0.75 wt.% of nanoparticles, the porosity of PP membrane increased from 31.48 % to 50.74 % decreasing its pore tortuosity. Addition of TiO_2_ nanoparticles could accelerate the crystallization rate and act as the crystal nuclei at the low quenching temperature [22], the average pore size and the porosity of nanocomposite membrane could be higher than those of the neat PP membrane.

The tensile strength of neat PP and nanocomposite membrane are shown in Table 1. According to the obtained results, the tensile strength of neat PP membrane increased by following the addition of nanoparticle. Similar to our previous findings [23], this manner can be attributed to the crystallinity change in PP and the reinforcement effect of the inorganic nanoparticles due to the addition of nanoparticle

### 3.3 Fouling analysis and membrane performance

**Fig. 3:**
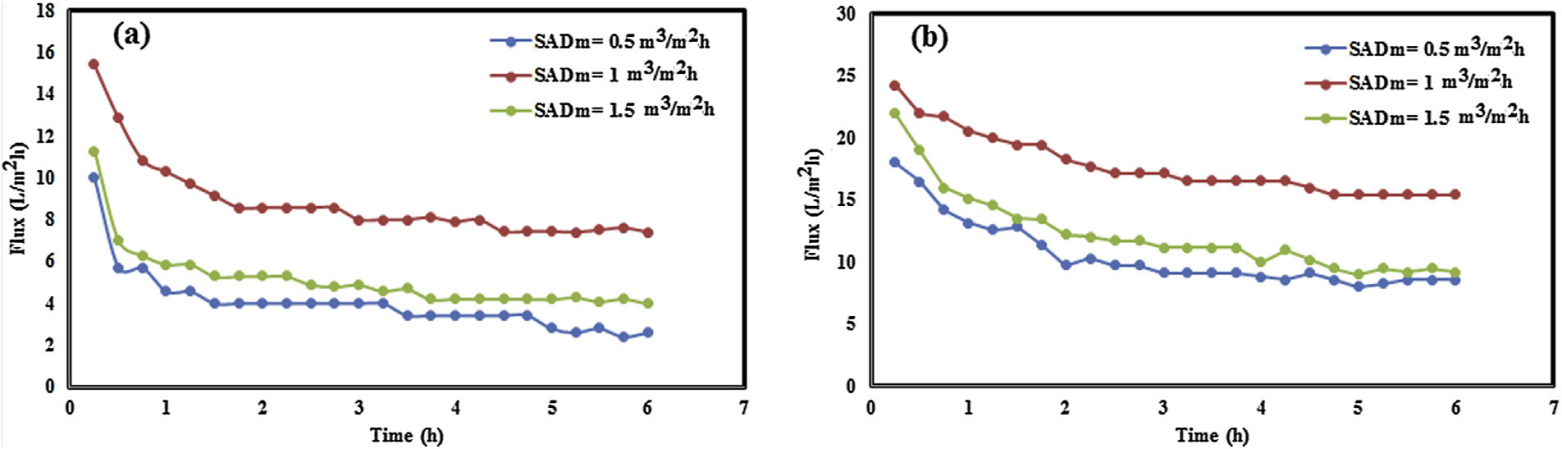
Flux decline with operation time for (a) neat PP and (b) PP/TiO_2_ membranes under different aeration rates and TMP = 0.1 bar.

**Fig. 4:**
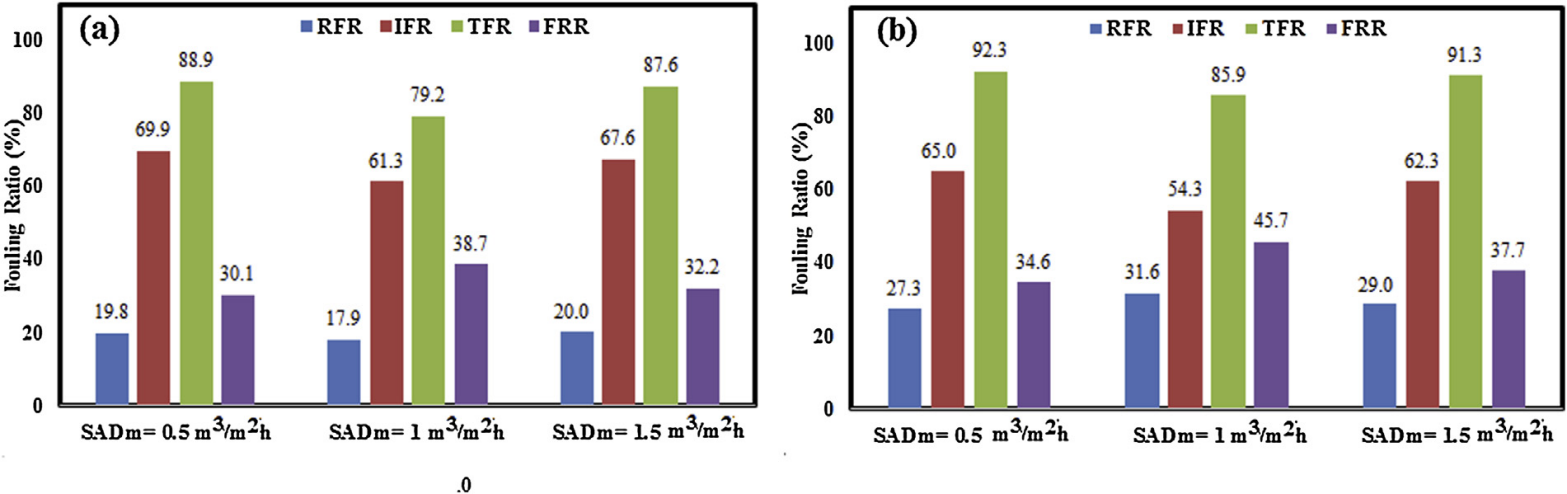
Effect of aeration rates on fouling parameters of (a) neat PP and (b) PP/TiO_2_ membranes.

In order to evaluate the effect of aeration rate on antifouling properties of neat PP and PP/TiO_2_ (0.75 wt.%) membrane, the permeate flux is plotted against time in Fig. 3 for various SADm, i.e. 0.5, 1, and 1.5 m^3^/m^2^h under TMP of 0.1 bar. As shown in Fig. 4a and b, for both membranes, flux at the end of filtration was decreased in the extreme low and high aeration rates. By increasing SADm from 0.5 to 1 m^3^/m^2^h and for both membranes, the flux through the membrane increased at the whole of permeation time. At higher aeration rates, i.e. 1.5 m^3^/m^2^h, membrane permeability decreased. The results confirm the importance of aeration as a means to mitigate fouling in immersed membrane systems.

By comparing Fig. 3a and b, it is clear that the in comparison with neat PP membrane and for all aeration rates, ﬂux has increased for nanocomposite membrane. That indicates that the membrane hydrophilicity and porosity as well as surface pore size (according to SEM images) played the vital role in the improvement of the activated sludge ﬂux.

Fouling analysis was made by calculating the reversible fouling ratio (RFR), irreversible fouling ratio (IFR), total fouling ratio (TFR), and ﬂux recovery ratio (FRR) of membranes under different aeration rates after activated sludge filtration test. These parameters are shown in Fig. 4. A higher FRR shows a better flux recovery while a lower IFR demonstrates a better performance controlling the total fouling [24]. In the aeration rate of 1 m^3^/m^2^h, the neat PP and PP nanocomposite membrane shows the lowest IFR and TFR among other aeration rates. The IFR values for neat PP membrane were calculated to be 69.9 % and 61.3 % when the SADm were 0.5 and 1 m^3^/m^2^h, respectively (see Fig. 4a). Also, the values for FRR were 30.1 %, 38.7 %, and 32.2 % for SADm of 0.5, 1, and 1.5 m^3^/m^2^h, respectively. Similar trend was found for PP nanocomposite membrane under different aeration rates (see Fig. 4b).

**Fig. 5:**
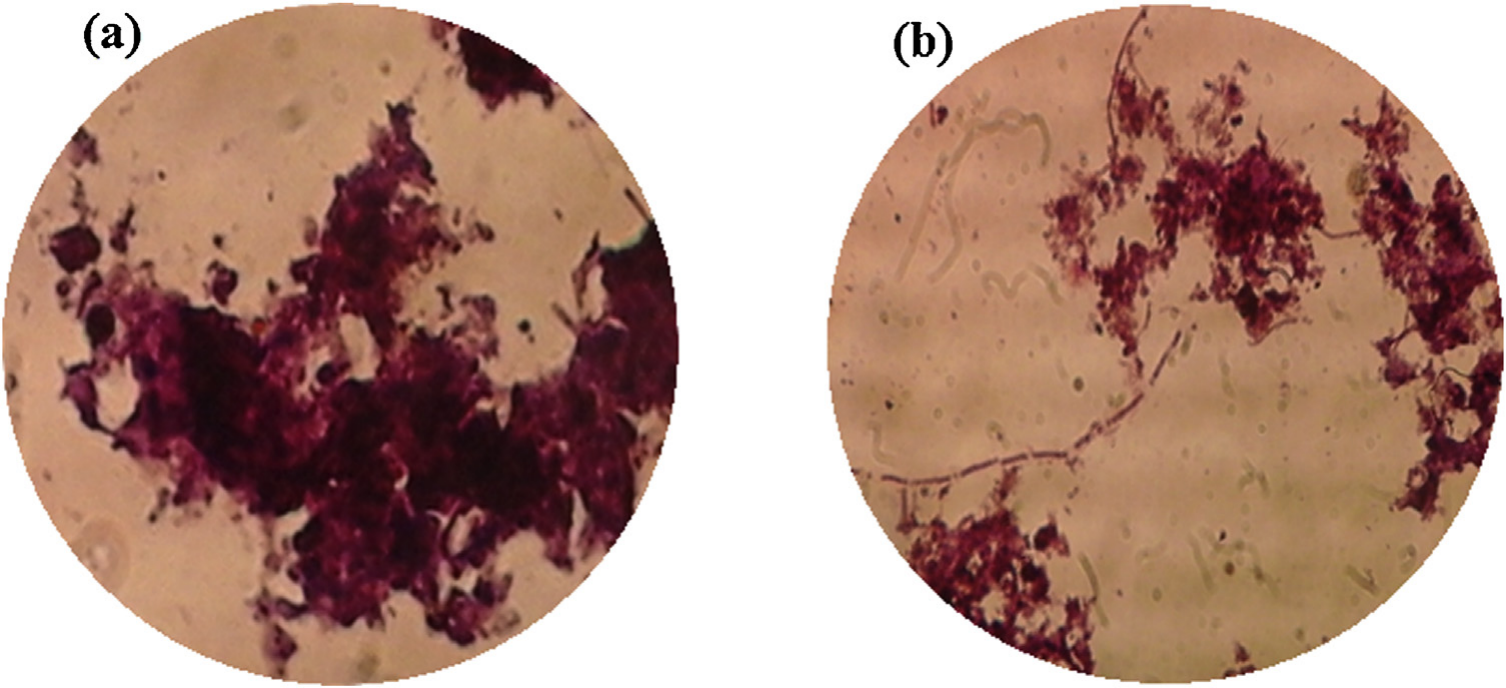
Microscopic images of sludge flocs in mixed liquor under: (a) lower (SADm = 0.5 m^3^/m^2^h) and (b) higher (SADm = 1.5 m^3^/m^2^h) aeration rates.

Higher aeration rates more efficiently remove the fouling deposition or cake layer on the membrane surface due to the higher shear force of bubble air, and simultaneously increases the breakage of components that have been identified as major contributors to fouling. Therefore, under high aeration rates, the membrane fouling intensifies. Meanwhile, the small matters from the occurrence of floc and particle breakage can penetrate the membrane pores, during which membrane pore blockage or irreversible fouling occurs. Fig. 5 shows the microscopic images of sludge flocs in mixed liquor under low (SADm = 0.5 m^3^/m^2^h) and high (SADm = 1.5 m^3^/m^2^h) aeration rates. It is clear that a low aeration rate results in larger floc and particles sizes, while a higher aeration rate creates smaller particle and flocs due to the floc breakage [25,26].

Comparing the RFR and IFR values of the neat PP and PP/TiO_2_ membranes however, shows that the RFR and IFR values of the nanocomposite membrane are higher and lower than the neat PP membrane, respectively, which confirms the improvement of the antifouling property of nanocomposite membrane due to hydrophilicity improvement. Generally, if the foulants (such as colloidal particles, sludge flocs and cell debris) are weakly bound on the membrane surface or within its pores, reversible fouling occurs, which can be easily eliminated by water rinsing. While, irreversible fouling occurs, when the foulants are strongly attached within the pores or membrane surface and chemical cleaning is seriously needed to remove these reagents [27,28].Therefore, it seems that reduction in IFR is important for membrane separation process due to chemical cleaning and subsequently resulting in high cost.

**Fig. 6:**
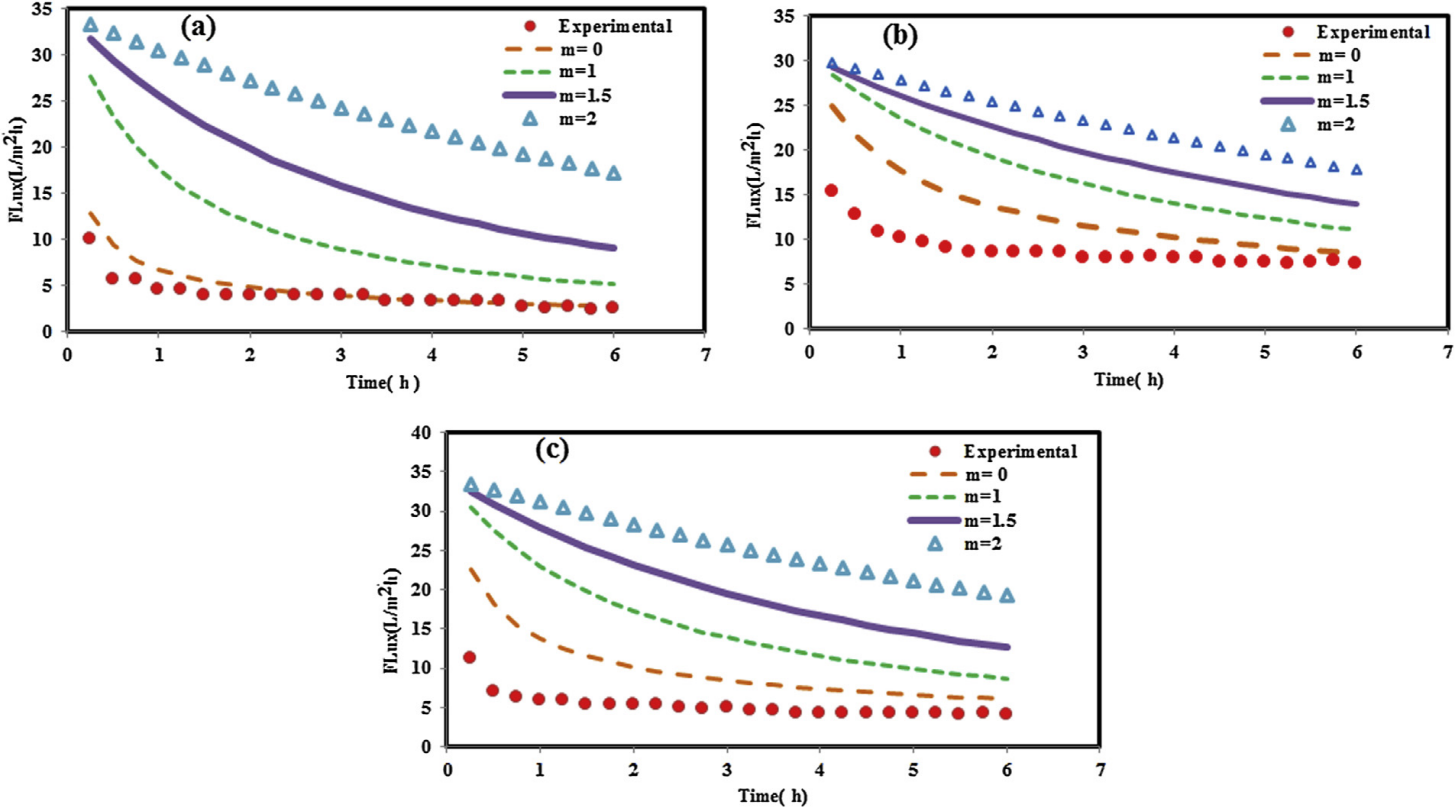
Experimental data and Hermia fouling models for neat PP membrane under different aeration rates: (a) SADm = 0.5 m^3^/m^2^h, (b) SADm = 1 m^3^/m^2^h, and (c) SADm = 1.5 m^3^/m^2^h.

Fig. 6 illustrates the fitting of the obtained experimental data after using the neat PP membrane in MBR system under various aeration rate conditions to different predicted fouling mechanisms, including complete pore blocking (m = 2), standard pore blocking (m = 1.5), intermediate pore blocking (m = 1), and cake formation (m = 0). In this study, in order to identify the mechanism of fouling during activated sludge filtration, the model k parameter was estimated by linear regression method. The adjusted values of k and correlation coefficient; R^2^, for m = 0, 1, 1.5, and 2 were used to solve the respective Hermia’s equations and the obtained results are shown in Table 2. According to Fig. 6a, it is observed that under lower aeration rate (SADm = 0.5 m^3^/m^2^h), a cake filtration model provides the best fit with neat PP membrane.

**Table 2:** Obtained k and correlation coefficient R^2^ values for Hermia fouling models under different aeration rates for neat PP and nanocomposite membranes.

Membranes SADm (m^3^/m^2^h)	m = 0		m = 1		m = 1.5		m = 2	
	k	R^2^	k	R^2^	k	R^2^	k	R^2^
0.5	0.021	0.947	0.038	0.867	0.037	0.841	0.151	0.783
PP 1	0.007	0.921	0.011	0.804	0.014	0.751	0.088	0.709
1.5	0.005	0.861	0.020	0.825	0.023	0.790	0.109	0.691
0.5	0.019	0.914	0.010	0.838	0.017	0.793	0.113	0.751
PP/TiO_2_ (0.75 wt.%) 1	0.004	0.879	0.004	0.792	0.008	0.687	0.069	0.682
1.5	0.003	0.870	0.009	0.841	0.017	0.801	0.122	0.770

**Fig. 7:**
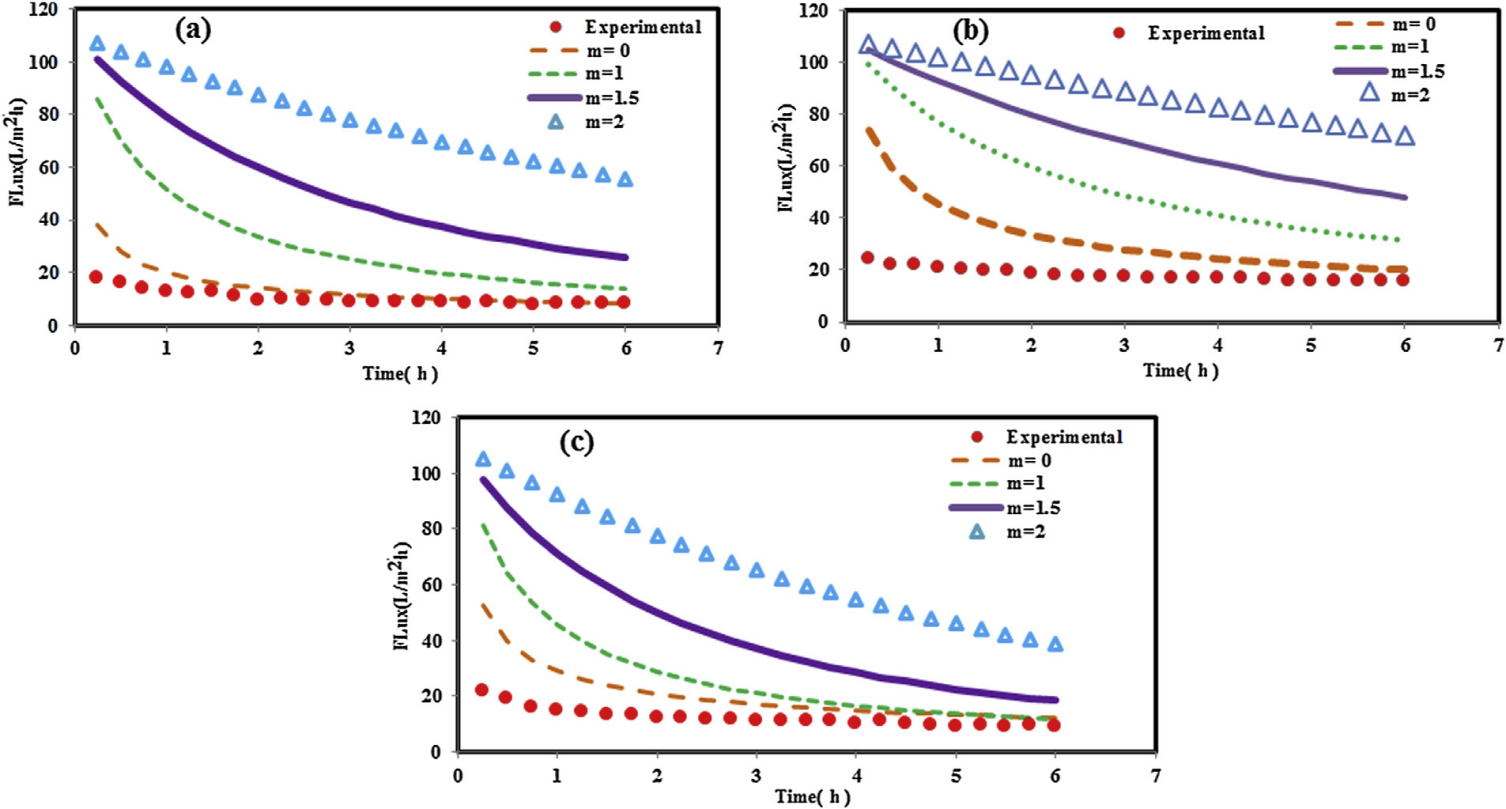
Experimental data and Hermia fouling models for PP/TiO_2_ nanocomposite membrane under different aeration rates: (a) SADm = 0.5 m^3^/m^2^h, (b) SADm = 1 m^3^/m^2^h, and (c) SADm = 1.5 m^3^/m^2^h.

In Hermia’s model, according to Zhang et al. [29] study, a k-value can be used to estimate the degree of membrane fouling. As shown in Table 2 and for neat PP membrane, for cake formation model, the increased k values under different aeration rates follow the following order: SADm = 1.5 m^3^/m^2^h < SADm = 1 m^3^/m^2^h < SADm = 0.5 m^3^/m^2^h, which indicates that the thickness of cake layer formed on the membrane surface under different aeration rates and for neat PP membrane is: SADm = 1.5 m^3^/m^2^h < SADm = 1 m^3^/m^2^h < SADm = 0.5 m^3^/m^2^h. Therefore, as shown in the Fig. 6, cake filtration mechanism is the dominant mechanism for neat PP membrane under lower aeration rate (SADm = 0.5 m^3^/m^2^h). Similar trend was found for PP/TiO_2_ nanocomposite membrane (see Fig. 7a).

As shown in Fig. 6, Fig. 7, by increasing in aeration rate from 0.5 to 1 and 1.5 m^3^/m^2^h, there is no model to quite well prediction of experimental data. These results indicated that the aeration rate largely determines the potential for floc breakage and release of small fragments into the bulk liquid which cause membrane pore blockage. As shown in Fig. 4, increasing in aeration rate (higher than SADm of 2 m^3^/m^2^h) results in irreversible fouling. Under lower aeration rate, due to larger size of sludge floc and other particles, these matters accumulated on the membrane surface and cake layer formed on the membrane surface which can easily remove physically or eliminated by water rinsing. However, under very high aeration rate (SADm = 1.5 m^3^/m^2^h) the floc and particle breakage occurs which these small maters can penetration through the membrane pores and membrane pore blockage or irreversible fouling occurs.

**Fig. 8:**
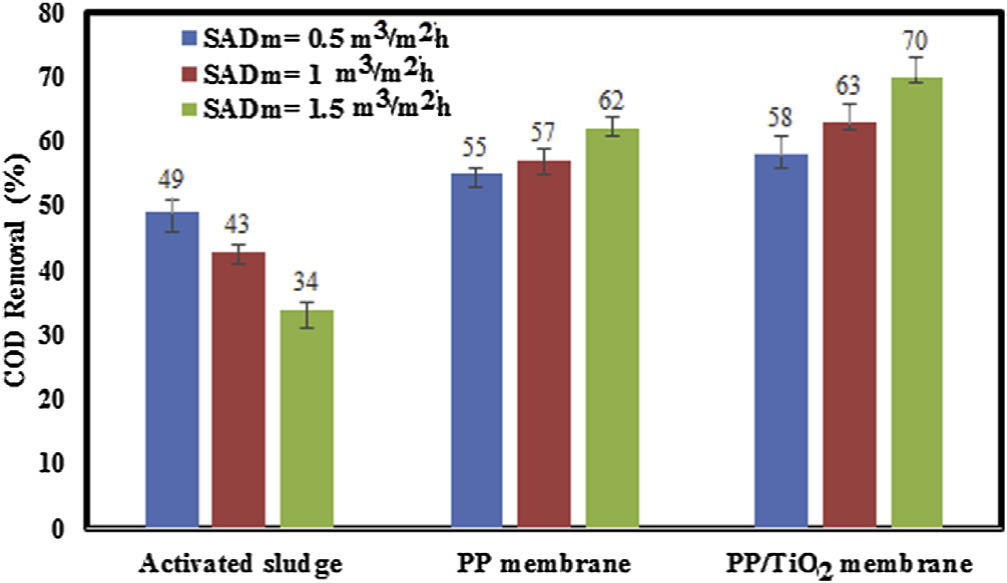
COD removal for activated sludge and membranes under different aeration rates.

**Fig. 9:**
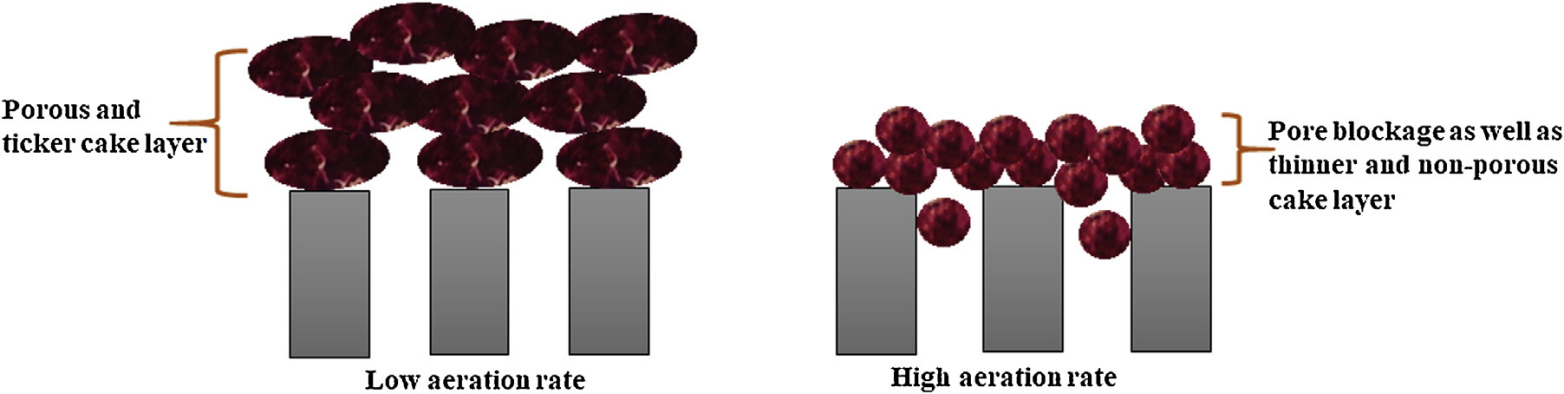
Schematic illustration of biofilm formation and pore blockage for membrane under lower and higher aeration rates.

The amount of COD removal for activated sludge and membranes under different aeration rates was also investigated and the obtained results were shown in Fig. 8. It can be observed that COD removal of activated sludge decreased by increasing aeration rate. According to the Meng et al. [10] and Temmerman et al. [25] studies, high aeration rates led to the release of soluble microbial products (SMP) and breakage of particles and bacteria. Therefore, it is expected that COD removal efficiency of activated sludge was decreased by increasing aeration rates. This trend was observed elsewhere [30]. However, COD removal increased by increasing aeration rate for neat PP and PP/TiO_2_ membranes. As shown in Fig. 8, the higher COD removal for both membranes was appeared at the highest aeration rate (SADm = 1.5 m^3^/m^2^h). It could be concluded that high aeration rate results in breakage of particles and bacteria, and as mentioned previously, under high aeration rate membrane pore blockage occurs and foulants cannot cross through the membrane and therefore membrane COD removal increased. In other words, under high aeration rate conditions, due to the floc breakage, membrane pore blockage as well as thinner and denser cake layer was formed on the membrane surface and this phenomenon acts as a secondary membrane that filters and prevents the penetration of foulants [31]. On the other hand, under lower aeration rate conditions, due to the presence of larger sludge floc in MBR tank, a thicker and porous cake layer was formed on the membrane surface which cause foulants cross a cake layer and membrane and subsequently results in increasing COD with respect to higher aeration rate. This phenomenon schematically is shown in Fig. 9.

Comparing the COD removal values for neat PP and PP/TiO_2_ membranes show that the COD removal for nanocomposite membrane was higher than neat membrane. This efficient reduction in COD can be ascribed to the presence of TiO_2_ nanoparticle which results in increasing in membrane hydrophilicity.

## 4 Conclusions

This study examined the effect of aeration rate on antifouling properties of polypropylene/TiO_2_ nanocomposite membrane in MBR system in order to oil refinery wastewater treatment. PP/TiO_2_ nanocomposite membranes with high hydrophilicity and high porosity were successfully fabricated via TIPS method. The obtained results indicated low or high aeration rate had a negative influence on membrane permeability. Low aeration could not remove the membrane foulants from membrane surface effectively. However, high aeration could induce a severe breakage of sludge flocs. The SADm of 1 m^3^/m^2^h was selected as optimal aeration rate for both neat PP and nanocomposite membranes, which low IFR and high FRR was occurred.

According to the results obtained from Hermia’s model, it can be determined that for both membranes, the best fit to experimental values are cake formation mechanism under lower aeration rate (SADm = 0.5 m^3^/m^2^h). However, by increasing aeration rate no one of models couldn’t predict experimental data. Also, the COD removal rates for activated sludge decreased as the aeration rate increased. While, by increasing aeration rate, COD removal for neat PP and PP/TiO_2_ membranes increased due to formation of pore blockage and denser cake layer. As a final result, PP nanocomposite membrane showed better antifouling properties compared to neat PP membrane.

## Declaration of Competing Interest

We have no conflicts of interest to declare.
